# Cost of Treatment in a US Commercially Insured, HIV-1–Infected Population

**DOI:** 10.1371/journal.pone.0098152

**Published:** 2014-05-27

**Authors:** Caitlyn T. Solem, Sonya J. Snedecor, Alexandra Khachatryan, Katherine Nedrow, Margaret Tawadrous, Richard Chambers, Seema Haider, Kit Simpson

**Affiliations:** 1 Pharmerit International, Bethesda, Maryland, United States of America; 2 Pfizer Inc, Groton, Connecticut, United States of America; 3 Pfizer Inc, Collegeville, Philadelphia, United States of America; 4 Medical University of South Carolina, Charleston, South Carolina, United States of America; Infectious Disease Service, United States of America

## Abstract

**Objective:**

Recent treatment patterns and cost data associated with HIV in the United States are limited. This study assessed first-line persistence and healthcare costs of HIV-1 in patients by treatment line and CD4 cell count.

**Methods:**

*MarketScan* Commercial Claims and Encounters Database (2007–2011) and Lab Database (2007–2010) were used to construct two HIV-1 cohorts: 1) newly treated HIV-1–infected patients with ≥6 months' continuous enrollment prior to first third-agent drug claim (Newly Treated Cohort) and 2) CD4 cell count test results (CD4 Measurements Cohort). All patients were ≥18 years old and without hepatitis co-infection. The Kaplan-Meier method was used to measure treatment switch rates. Generalized linear models (gamma distribution, log link) were used to compare healthcare costs by treatment line and CD4 cell count controlling for potential confounders.

**Results:**

Newly treated patients (n = 8,617) had mean age of 41, 82% were male, and 20% had experienced AIDS-defining events at baseline. Over 20% of newly treated patients switched initial treatment regimen within 2 years. Average unadjusted (and covariate-adjusted) total healthcare cost/year was $33,674 ($28,861) for first-line, $39,191 ($35,805) for second-line, and $39,882 ($40,804) for third-line treatment. Covariate-adjusted costs of care on second- and third-line treatments were significantly more expensive than first-line treatment (24% [p<0.001] and 41% [p = 0.006] higher, respectively). The CD4 Measurements Cohort included 803 CD4 measurements (mean age 49, 76% male, 8% experienced an AIDS-defining event). Costs associated with CD4 measurements <100 cells/µL were 92% higher than those with >350 cells/µL (p<0.001). For higher CD4 cell counts, the majority of expenditures were for antiretrovirals (64% of total for CD4 >350 cells/µL).

**Conclusions:**

Despite modern advances in antiretroviral therapy and medical care, direct medical costs of HIV-1–infected patients increase after treatment switch and with lower CD4 counts, consistent with previous costing studies.

## Introduction

Human immunodeficiency virus (HIV) infections remain prevalent within the United States (US), where approximately 1.26 million people are afflicted and 50,000 new infections occur annually [1], [2]. The virus replicates in and kills CD4-expressing white blood cells, causing them to gradually decrease in number, ultimately leading to Acquired Immunodeficiency Syndrome (AIDS), where a patient is unable to fight off infections and disease. Modern antiretroviral (ARV) drug regimens effectively suppress virus replication, allowing patients to maintain higher levels of CD4 cells and delay the progression to AIDS. These treatments have increased the life expectancy for patients with HIV infection from 6–10 years to values approaching those of the general population [3], [4].

While effective, ARV treatment does not represent a cure for HIV infection, and treatments must be taken for the duration of a patient's lifetime. Patients are typically treated with a combination of drugs, including at least two nucleoside reverse transcriptase inhibitors (NRTIs) and at least one drug from the other, more potent drug classes (commonly called the “third-agent” treatment). Third agents are treatments from the non-NRTI, protease inhibitor, and integrase inhibitor drug classes and are responsible for the large improvement in the treatment of HIV disease by combination therapy observed in the last 2 decades. These combinations are costly; the most popular combination (efavirenz/emtricitabine/tenofovir) is published to have an average wholesale price of $2,253.88 per 30 tablets [5]. Despite the high cost of ARVs, studies have shown early combination treatments to be cost-effective [6] because they reduce the economic burden of illness associated with AIDS-related hospitalizations and death and increase patients' quality of life. Thus, HIV disease has created a large economic burden, with annual costs of care for HIV-infected patients in the US estimated to be $19,912 in 2006 dollars [7] and discounted lifetime costs estimated at $385,200 (2004 dollars) [8].

Treatment switching is often the result of intolerance or virologic failure (the inability of ARVs to suppress circulating virus to undetectable limits or rebound of virus after previous suppression). In the case of the latter, future regimens may be less effective [9] and thus could result in higher associated healthcare costs. Longer duration of virologic suppression has also been associated with decreased risk of subsequent virologic failure [10]. Therefore, use of safe, well-tolerated, and effective anti-HIV regimens is paramount to allow patients to remain on first-line treatment for as long as possible, which may lead to improved economic and clinical outcomes, including immune function, quality of life, and ability to control other comorbid conditions (e.g., hepatitis C co-infection) after adequately controlling HIV [11], [12].

Recent estimates of the economic burden of HIV disease and costs of treatment switching are not available. The most recent literature reporting cost data associated with HIV in the US reflects costs by CD4 cell count prior to 2006 [8] and costs by treatment line in 2000–2004 [13]. These estimates may not reflect the costs of current ARV therapies or advancements in other healthcare treatments [14]. This study assessed current first-line ARV persistence and healthcare costs of HIV-1–infected patients by treatment line (i.e., first, second, and third line) and by CD4 cell count, using a US commercial claims database. Understanding the current costs of care provides insight into the economic benefits and consequences of currently used ARV treatments.

## Methods

### Sample Selection

The *MarketScan* Commercial Claims and Encounters Database (2007–2011) and the *MarketScan* Lab Database (2007–2010) (Truven Health Analytics, Ann Arbor, MI) were analyzed to determine total costs for HIV-1–infected patients. These data sets include claims compiled from 100 employers and 12 health plans throughout the US, representing more than 30 million covered lives. Patients with any HIV-1 diagnosis code (ICD-9-CM 042, V08, or 795.71) in any inpatient, outpatient, or service claim between January 1, 2007, and December 31, 2011, who were ≥18 years of age and had no comorbid diagnosis codes for HIV-2 (ICD-9-CM 079.53) or hepatitis C (ICD-9-CM 070.41, 070.44, 070.51, 070.54, 070.70, 070.71, or V02.54) were considered for inclusion in the analysis. From these data, two patient cohorts were constructed: 1) Newly Treated Cohort—patients initiating ARV treatment, including a third-agent drug and at least one NRTI; and 2) CD4 Measurements Cohort—patients with one or more CD4 cell count test result data.

**Figure 1:**
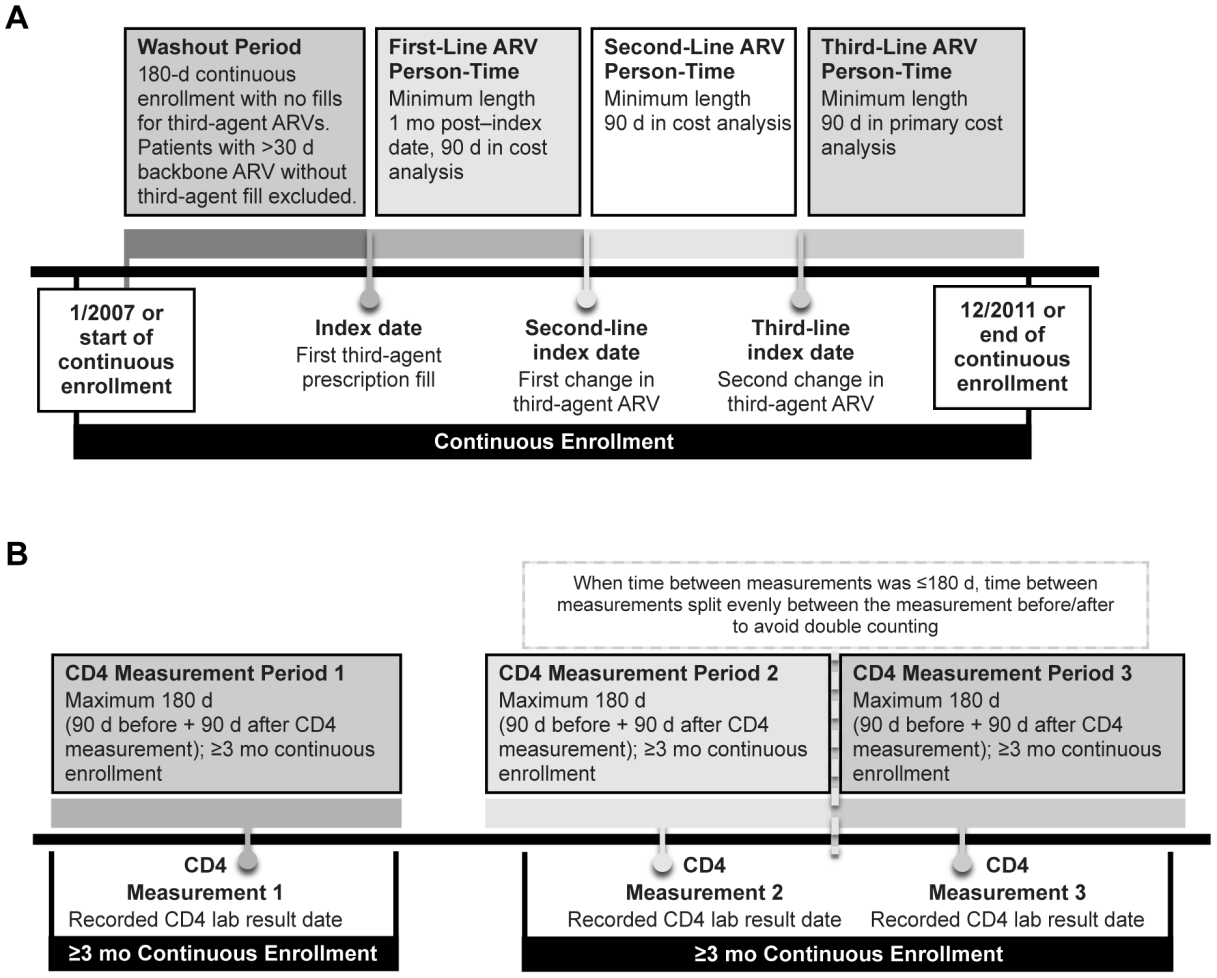
Patient selection: (A) Newly Treated Cohort; (B) CD4 Measurements Cohort. ARV, antiretroviral.

Patients included within the Newly Treated Cohort were those with ≥6 months' continuous enrollment prior to the first claim for a third-agent ARV (e.g., non-nucleoside reverse transcriptase inhibitor, protease inhibitor, fusion inhibitor, integrase inhibitor), ≤30 days of NRTI use prior to first third-agent claim, at least 1 month of continuous enrollment after first third-agent claim, and use of at least one NRTI during the follow-up period (Figure 1A). The patient's first claim for a third agent served as the patients' index date, and patients were followed until the end of their continuous enrollment or the last date of follow-up (i.e., December 30, 2011).

Each CD4 measurement was a unique observation defining the CD4 Measurements Cohort, with the date of CD4 measurement serving as the index date (Figure 1B). To allow sufficient follow-up time to determine costs, measurements also had to include ≥90 days of continuous enrollment pre- and/or post-measurement, with a maximum of 90 days before and 90 days after the index date.

### Baseline Patient Characteristics

Patient characteristics included age, geographic region, health plan type, and gender, as available. In addition, categories of comorbidities were defined, and the Charlson Comorbidity Index (defined using Charlson-Deyo algorithm [15]) and occurrence of AIDS-defining events were measured prior to the index date. AIDS-defining events included ≥1 instance of an ICD-9-CM code for any of the following conditions: candidiasis, herpes simplex, wasting syndrome due to HIV, encephalopathy, recurrent pneumonia, lymphoma, Kaposi's sarcoma, cytomegalovirus, pneumocystis, pneumonia, tuberculosis, cryptococcosis, coccidioidomycosis, toxoplasmosis of the brain, mycobacterium, histoplasmosis, salmonella, isosporiasis, or progressive multifocal leukoencephalopathy [16].

### Determination of Treatment Lines

Lines of treatment were described in the Newly Treated Cohort. Treatment switch to second or third line was defined as a change to the third agent of the regimen, among treatments where only one third agent was used. In the context of treatment switch, regimens containing more than one third agent were considered “other” and were not distinguished from each other. Treatment switches involving only the NRTI backbone within a regimen were not considered.

### Cost of Care

Total treatment costs included costs of all patient claims, including inpatient, outpatient, emergency room, and prescription claims (stratified into ARV and non-ARV costs). Cost outcomes were standardized into monthly units within the CD4 Measurements Cohort (costs per person-month  =  sum of costs observed over CD4 measurement period x 30 divided by number of days in CD4 measurement period) and yearly units in the Newly Treated Cohort (costs per person-year  =  all costs observed over the period within a treatment line x 365 divided by number of days in the period of the treatment line). All cost data were inflated to 2011 US dollars using the Bureau of Labor Statistics Consumer Price Index for medical care [17].

Cost analysis for the Newly Treated Cohort was limited to treatment lines ≥90 days. Without patients' viral load data, it was not possible to identify whether the reason for treatment switch was due to intolerance, virologic failure, or other. One could assume that treatment switching within 90 days would likely be due to reasons of intolerance and the subsequent regimen's persistence would not be affected by the switch. For this reason and to allow for sufficient time for cost accrual, the cost results are restricted to only treatment lines ≥90 days in length. Within the CD4 Measurements Cohort, results are presented for measurement periods with ≥1 ARV prescription to minimize the effect of missing ARV cost data due to the possibility of patients receiving treatment outside of their health plans.

### Statistical Analyses

Analyses to address the study objectives were primarily descriptive in nature. Age data were presented as means and standard deviations along with medians and interquartile ranges. Age by categorical strata, gender, health plan type, geographic region, Charlson Comorbidity Index, and presence of AIDS-defining conditions were described using proportions. The Kaplan-Meier method was used to estimate probability of initial treatment switch accounting for the presence of censored data due to different lengths of follow-up time.

Because cost data are always positive and not normally distributed, generalized linear models with a gamma distribution and log link were used to compare healthcare costs by treatment line and CD4 cell count controlling for potential confounders including Charlson comorbidity index, presence of AIDS-defining events, sex, age, region (Newly Treated Cohort only), and type of health plan. From these models, adjusted mean costs were calculated for treatment line and CD4 cell count strata using the coefficients from the generalized linear model, setting all other parameters within the model to their mean value. Unadjusted mean costs were calculated as simple means. Percentage of costs attributable to different sources (inpatient, outpatient, ARV drug, non-ARV drug, ER, other) was similarly modeled to control for covariates. Due to the high number of zeroes for inpatient and ER costs, a two-part model was created. First, the probability of having a non-zero cost, adjusted for covariates, was estimated by logistic regression. Second, this probability was multiplied by the adjusted mean cost estimated by a generalized linear model of the observed non-zero costs. Sensitivity analyses were conducted for cost models by including treatment lines <90 days in length within the Newly Treated Cohort and including CD4 measurements without an ARV prescription fill in the measurement window within the CD4 Measurements Cohort.

## Results

### Study Population

**Figure 2:**
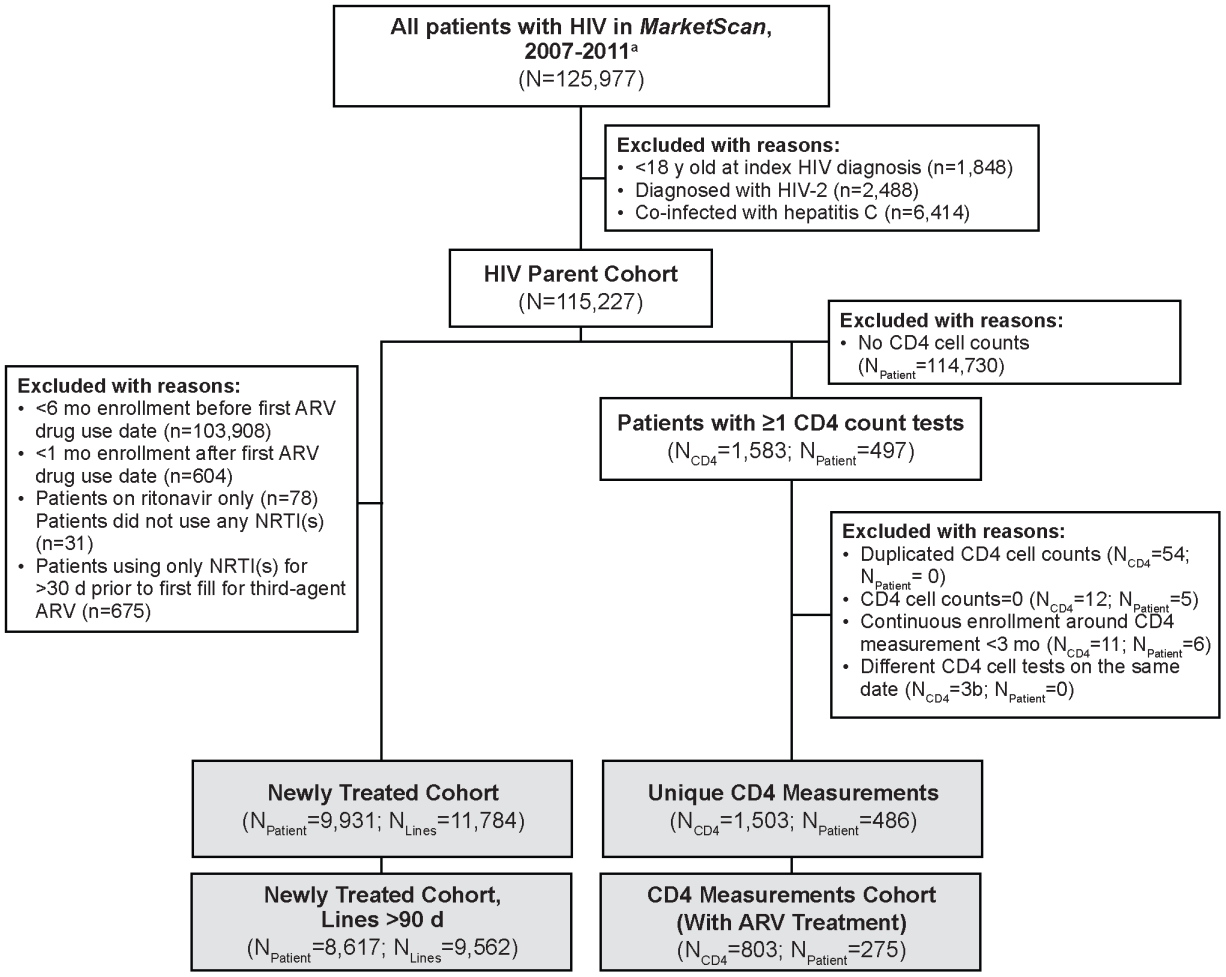
Flow chart of cohort selection. ARV, antiretroviral; NRTI, nucleoside reverse transcriptase inhibitor. ^a^Laboratory data provided through 2010. ^b^Three pairs of CD4 cell tests were combined by using the average laboratory result on the same date.

Of 125,977 patients with any HIV diagnosis within the *MarketScan* database during the study intake period (January 1, 2007, to December 31, 2011), a total of 9,931 patients met inclusion criteria for the Newly Treated Cohort (Figure 2), of which 8,617 had at least one treatment line with a duration ≥90 days. Patients within the Newly Treated Cohort had a mean age at the patient's index date of 40.5 years, 82.3% were male, and half of the patients (51.2%) were located in the South geographic region. Few patients (23.1%) had comorbid conditions, and 20.4% had experienced AIDS-defining events prior to starting third-agent treatment (Table 1).

**Table 1:** Demographic and Clinical Characteristics, by Cohort.

	Newly Treated Cohort, lines ≥90 d (N = 8,617 patients)	CD4 Measurements Cohort (N = 803 measurements)
**Age in y**, mean (SD)	40.5 (10.1)	48.6 (8.1)
Median (IQR)	41.0 (33.0–48.0)	49.0 (44.0–54.0)
**Age**, No. (%)		
18–34 y	2515 (29.2%)	43 (5.4%)
35–44 y	2976 (34.5%)	176 (21.9%)
45–54 y	2393 (27.8%)	397 (49.4%)
≥55 y	733 (8.5%)	187 (23.3%)
**Gender—male**, No. (%)	7088 (82.3%)	610 (76.0%)
**Health plan**, No. (%)		
Point of service	984 (11.4%)	17 (2.1%)
Health maintenance organization	1642 (19.1%)	57 (7.1%)
Preferred provider organization	4940 (57.3%)	593 (73.8%)
Comprehensive coverage	105 (1.2%)	0
Other^a^	946 (11.0%)	136 (16.9%)
**Region**, No. (%)		
Northeast	1123 (13.0%)	758 (94.4%)
North central	1313 (15.2%)	0 (0)
South	4415 (51.2%)	32 (4.0%)
West	1554 (18.0%)	12 (1.5%)
Unknown	212 (2.5%)	1 (0.1%)
**Charlson Comorbidity Index**, No. (%)		
0	6625 (76.9%)	702 (87.4%)
1	1165 (13.5%)	59 (7.4%)
2	495 (5.7%)	22 (2.7%)
≥3	332 (3.9%)	20 (2.5%)
**Any AIDS-defining conditions**, No. (%)	1760 (20.4%)	116 (14.4%)

IQR, interquartile range; SD, standard deviation.

a Other health plan includes basic/major medical, exclusive provider organization, consumer-directed health plan, and high-deductible health plan.

After matching patients who met study inclusion criteria to the *MarketScan* Laboratory data, only 486 patients had any CD4 cell measurements recorded, with a total of 1,503 unique CD4 cell counts. Of these measurements, 803 contained at least one ARV prescription fill within the window of measurement (Figure 2). The majority of measurements came from patients located in the Northeast (94.4%); 87.4% of patients had no comorbid conditions, and 7.7% had experienced an AIDS-defining event prior to their CD4 measurement (Table 1).

### Treatment Switches

**Figure 3:**
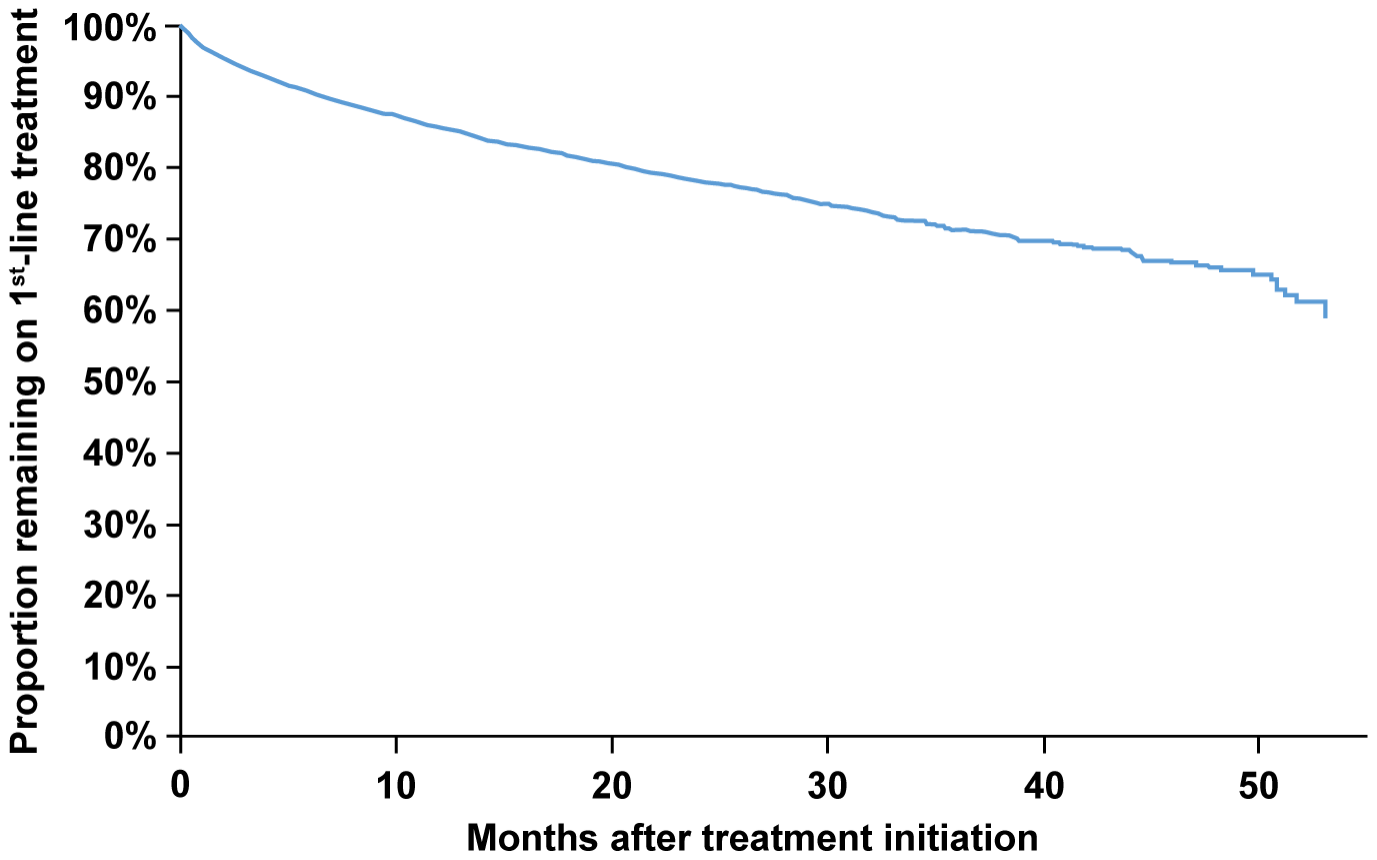
Kaplan-Meier estimate of time to first treatment switch.

Out of 9,931 patients in the Newly Treated Cohort, 1,540 (15.5%) were observed to switch from first line to a second line of treatment in 132,487 months of patient follow-up. Of those, 108 (7.0%) had switched to a third line of treatment during the observation period. Kaplan-Meier estimates accounting for censored follow-up times predicted 6.1%, 9.3%, 14.4%, and 22% of the patients on first-line treatment switched treatment at 3 months, 6 months, 12 months, and 24 months, respectively (Figure 3).

### Costs

**Figure 4:**
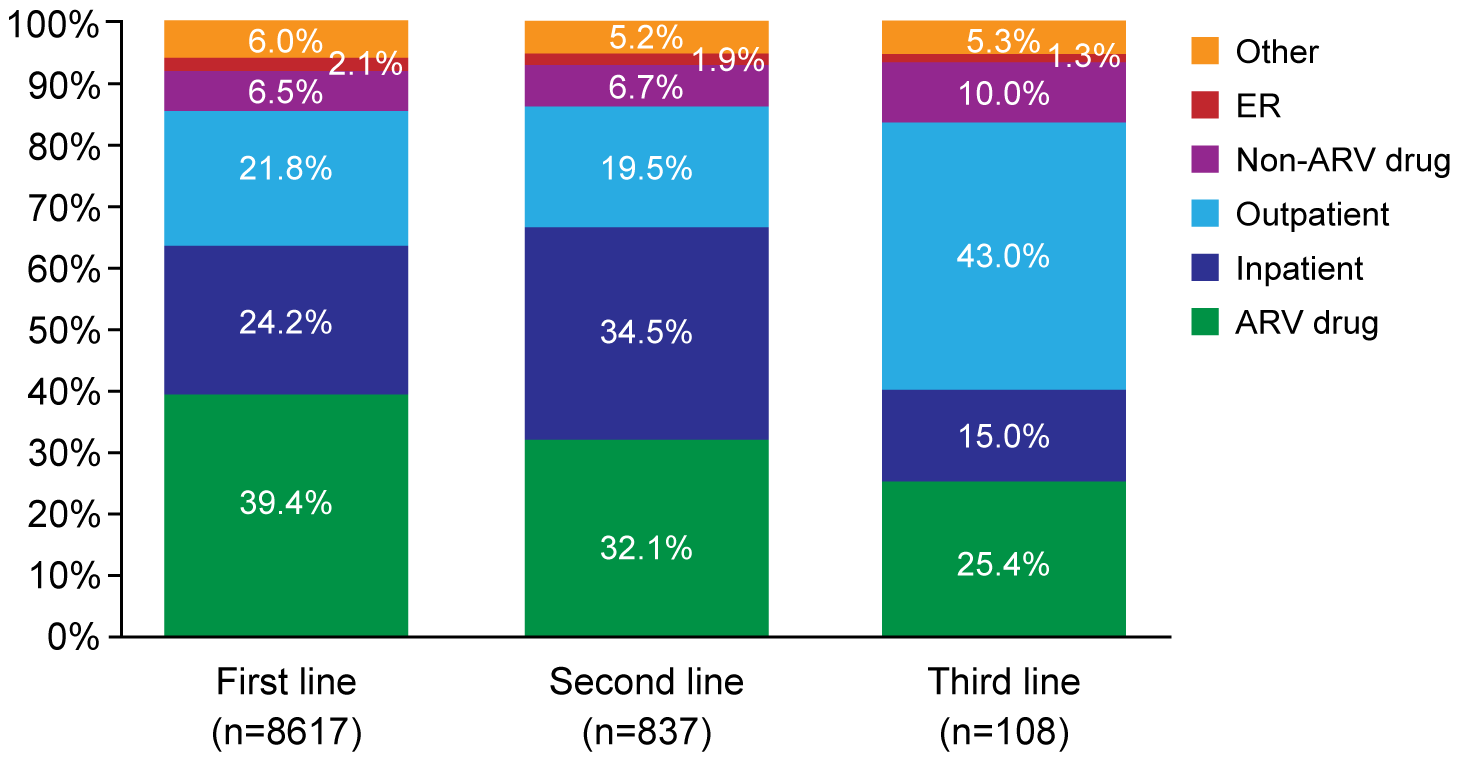
Proportion of total cost by component and treatment line in the Newly Treated Cohort. ARV, antiretroviral; ER, emergency room.

Within the Newly Treated Cohort, when considering lines of treatment ≥90 days long, unadjusted total mean costs per person-year increased with each subsequent treatment line: $33,674 for first line, $39,191 for second line, and $39,882 for third line. After adjusting for covariates, second-line was 24% (p<0.001) and third-line was 41% (p = 0.006) more expensive than first-line treatment (Table 2). Adjusted mean costs were $28,861 (95% CI: 28,051–29,695) for first-line, $35,805 (95% CI: 32,756–39,137) for second-line, and $40,804 (95% CI: 31,867–52,246) for third-line treatment per person-year. Within the Newly Treated Cohort, results were consistent when lines <90 days were included (second line 27% and third line 32% more costly than first line). The greatest driver of costs was ARV drug treatment, representing 39.4% of total costs in first-line, 32.1% in second-line, and 25.4% in third-line treatment. The next-largest drivers of costs were inpatient and outpatient medical utilization (Figure 4).

**Table 2:** Adjusted Models for Total Costs by Treatment Line and CD4 Cell Count.

	Newly Treated Cohort		CD4 Measurements Cohort^a^	
	Cost ratio (95% CI)	p value	Cost ratio (95% CI)	p value
**Treatment line (ref: first line)**				
Second line	1.24 (1.13–1.36)	<0.001		
Third line	1.41 (1.10–1.81)	0.006		
**CD4 cell count category (ref: >350 cells/µL)**				
<100 cells/µL			1.92 (1.34–2.76)	<0.001
100–350 cells/µL			0.94 (0.85–1.05)	0.264
**Charlson Comorbidity Index (ref: no comorbidities)**				
1	1.44 (1.38–1.50)	<0.001		
2	2.17 (2.03–2.32)	<0.001		
≥3	3.54 (3.28–3.83)	<0.001		
Any comorbidity			1.10 (0.95–1.26)	0.195
**Any AIDS-defining events**	1.69 (1.61–1.76)	<0.001	1.18 (0.99–1.40)	0.060
**Female**	0.86 (0.83–0.89)	<0.001	0.88 (0.80–0.98)	0.018
**Age (ref: 18–34 y)**				
35–44 y	1.48 (1.42–1.53)	<0.001	1.15 (0.94–1.42)	0.179
45–54 y	1.55 (1.49–1.61)	<0.001	1.35 (1.11–1.65)	0.003
≥55 y	1.76 (1.67–1.85)	<0.001	1.58 (1.28–1.95)	<0.001
**Region (ref: South)**				
North central	0.88 (0.85–0.92)	<0.001		
Northeast	0.67 (0.64–0.69)	<0.001		
Unknown	1.04 (0.93–1.17)	0.447		
West	0.91 (0.87–0.96)	<0.001		
**Health plan (ref: PPO)**				
Comprehensive	1.15 (1.03–1.29)	0.015	b	
HMO	1.15 (1.10–1.20)	<0.001	0.83 (0.70–0.98)	0.032
Other^c^	1.05 (1.00–1.10)	0.040	0.74 (0.66–0.83)	<0.001
POS	0.91 (0.87–0.96)	<0.001	0.98 (0.72–1.33)	0.889

ARV, antiretroviral; CI, confidence interval; HMO, health maintenance organization; POS, point of service; PPO, preferred provider organization.

a Model additionally controlled for the degree to which patients were exposed to ARV prior to the index CD4 measurement (filled prescription for a third agent, exposed to backbone treatments but not third agent, no ARV exposure prior to index).

b No CD4 measurements occurred in patients with comprehensive care.

c Other health plan includes basic/major medical, exclusive provider organization, consumer-directed health plan, and high-deductible health plan.

**Figure 5:**
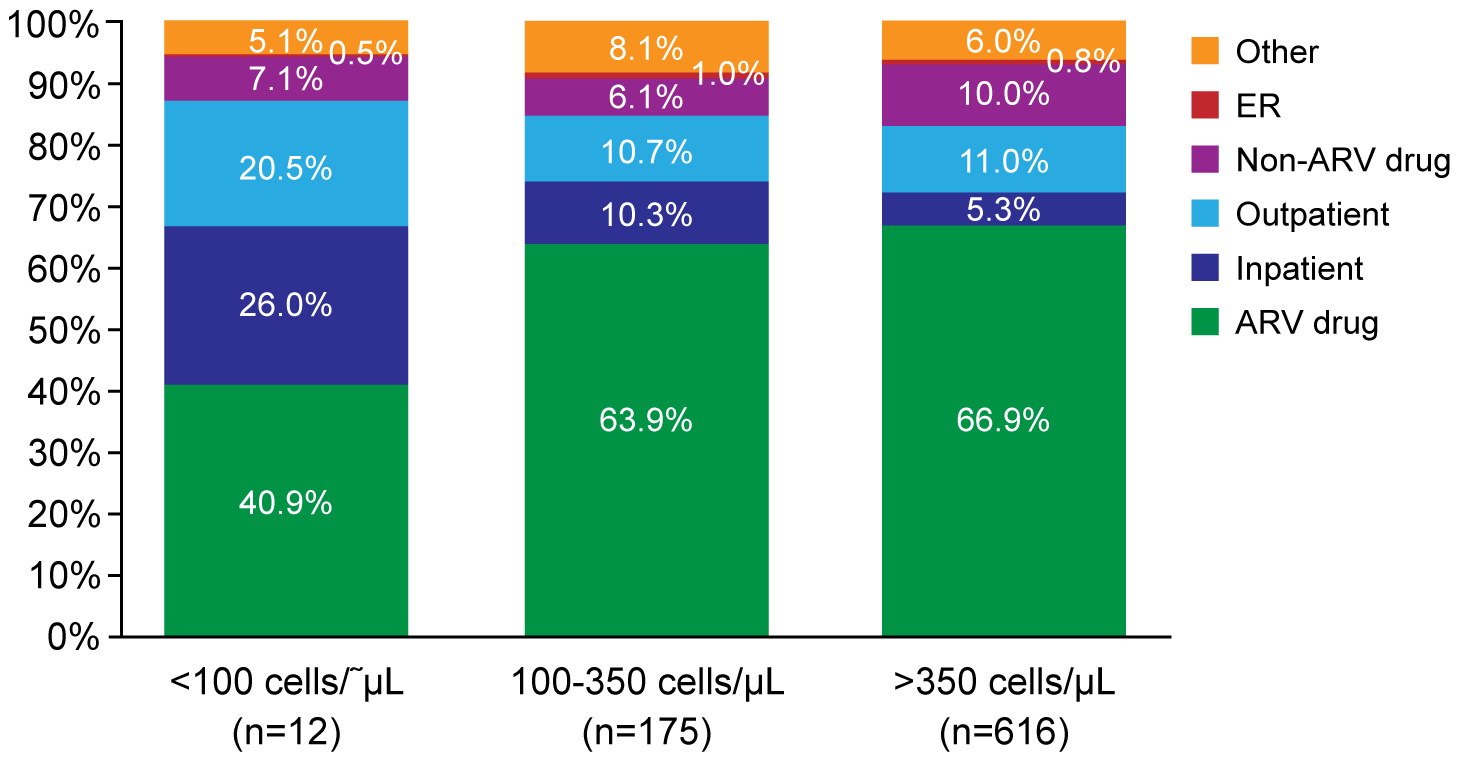
Proportion of total cost by component and CD4 stratum in the CD4 Measurements Cohort with ARV treatment. ARV, antiretroviral; ER, emergency room.

Within the CD4 Measurements Cohort, inpatient admissions became an increasing cost driver at lower CD4 cell counts (Figure 5). Unadjusted total mean costs per person-month were $5573, $2441, and $2631 for patients with CD4 cell counts <100, 100 to 350, and >350 cells/µL, respectively. After adjustment for prior ARV exposure, comorbidities, AIDS-defining events, sex, age, and health plan type, patients with CD4 cell counts <100 cells/µL had significantly higher (92% increased; p<0.001) costs per person-month as compared to those with CD4 cell counts >350 cells/µL (Table 2). Adjusted mean costs were $4,860 (95% CI: 3,401–6,945) for CD4 cell counts <100 cells/µL, $2,378 (95% CI: 2,166–2,611) for CD4 cell counts between 100–350 cells/µL, and $2,526 (95% CI: 2,405–2,654) for CD4 cell counts >350 cells/µL per person-month. When all CD4 measurements, including those that did not have an ARV fill within the measurement window, were modeled, costs associated with CD4 cell counts from 100–350 cells/µL were also significantly more costly compared to those with CD4 cell counts >350 cells/µL.

## Discussion

This study identified significantly higher healthcare costs associated with treatment switches and lower CD4 cell counts within a current managed-care claims database reflecting modern-era treatments. We also identified that 22% of newly treated patients switched third-agent treatments within 2 years, confirming that despite the increase in the potency and number of HIV treatments available, more than one-fifth of patients do not achieve long-term persistence on their initial regimens and move to second-line regimens, where the costs of treatment seem to increase.

These estimates were obtained from a retrospective analysis of a database including cost years that are more recent than what is currently available in the published literature and are most generalizable to HIV-1–infected patients who are covered by commercial insurance, are under the age of 65, and who do not have comorbid hepatitis C infection. Other patients not included within these parameters may have lower or higher costs due to lack of access to medical care (e.g., if uninsured) or additional health care requirements to manage comorbidities (e.g., if infected with hepatitis C or >65 years old). Older database analyses have shown healthcare costs to be higher for patients with lower CD4 cell levels. Two studies reported mean annual costs of patients with <50 cells/µL to be $35,000–$40,000, while patients with >350 cells/µL were estimated to have annual costs of approximately $15,000 (2000 [18] and 2006 [7] USD). Healthcare costs measured from 1996–1998 demonstrated that monthly costs significantly increased by $1,400 for one and $1,900 for more than one loss of virologic suppression [19]. Another study estimated the marginal cost of total care to be $35,000 higher during a 60-month follow-up period for patients on third- or greater line treatment compared with those on first- or second-line treatment (cost year not reported) [20]. Results obtained here confirmed that costs of CD4 cell decline and treatment switching continue to increase even in the era of modern ARV and medical treatment.

The *MarketScan* database provides what is perhaps the most clinically rich, commercially available data set for costs stratified by CD4 cell count. However, it has a few limitations. The CD4 laboratory data were primarily from a small proportion of total patients who lived in the Northeast region (94.4%), which may limit generalizability of these results to the entire population if differences exist in treatment practices and associated costs across regions. The database was limited in the availability of lab results for patients outside of the Northeast region. The limited geography among patients with CD4 cell measurements and the fact that all patients in our sample had healthcare insurance and access to medical care also contributed to the small number of (patients with) measurements in the lower CD4 stratum (12 out of 803 with <100 cells/µL).

Due to complex treatment patterns within the Newly Treated Cohort, we were able to select patients with concurrent use of NRTIs alongside their third-agent treatments but were unable to ensure that these patients were consistently using at least two NRTIs, which would be consistent with clinical guidelines for appropriate HIV treatment. However, given that the US guidelines regarding multiple NRTI use within combination treatment regimens have existed for nearly 20 years, the likelihood that any patient in our sample used only one NRTI is probably low.

Although costs by CD4 cell level and line of treatment were measured within the same database, these two estimates are possibly correlated. As patients progress through lines of treatment, their CD4 cells may concurrently decline, an important confounder in cost estimates. Because of the limited CD4 cell data available, there was very little patient overlap between the Newly Treated and the CD4 Measurements Cohorts (n = 29), precluding any adjustment of the treatment line costs for CD4 cell level. A data set based on a chart review or other clinical records may be necessary to properly estimate costs of treatment switching while adjusting for reasons for switch and CD4 cell levels. This is evidenced by the increasing proportion of non-ARV drug costs (Figure 4), which could indicate a worsening of condition with later lines of treatment.

A limitation typical of all retrospective claims database analyses is that cost data were limited to medical care received within plans included in *MarketScan* and may have underestimated costs for patients who receive supplemental healthcare not included within the claims database. This is a particular problem for estimating the costs of HIV in the US, where AIDS Drug Assistance Programs provide treatment to approximately one-third of infected patients [21]. By requiring all CD4 cell measurement periods to contain at least one ARV claim within our primary analyses, we aimed to minimize the impact of missing drug cost data, although we cannot rule out the possibility of misclassification of Newly Treated Cohort patients who had previously received ARVs outside of their health plans.

To obtain an analysis as close to the “real world” as possible, we restricted the primary CD4 cell measurement analyses to be representative of expected treatment patterns and the treatment line analyses to be representative of switching for reasons of treatment failure. These results confirmed that HIV infection continues to be a costly disease even in the era of modern ARV treatments and provided a recent estimate of costs by treatment line and CD4 cell level, filling a gap in the US economic literature. Our analysis showed that during the years 2007 through 2011, US patients receiving first-line treatment had significantly lower total healthcare costs than patients receiving second-line or third-line therapies. This indicates that further investment to develop additional treatment regimens to suppress viral replication and preserve patients' CD4 cells through multiple lines of treatment are fundamental to long-term HIV disease management and reduction of the economic burden of advanced HIV disease.
